# HANDS AND HEALTH AT HOME: THE IMPACT OF INTERGENERATIONAL SERVICE-LEARNING ON STUDENTS’ PREHEALTH COMPETENCIES

**DOI:** 10.1093/geroni/igae098.0173

**Published:** 2024-12-31

**Authors:** Anna Schwartz, Rachel Logue Cook, Courtney Vanderlaan, Susan Brown

**Affiliations:** University of Michigan, Ann Arbor, Michigan, United States; University of Michigan, Ann Arbor, Michigan, United States; Michigan Medicine, Ann Arbor, Michigan, United States; University of Michigan, Ann Arbor, Michigan, United States

## Abstract

There is a critical need for developing the geriatric healthcare workforce, yet pre-health students are rarely given the opportunity to engage with older adults during their coursework. In addition, many health-related professional programs require students to demonstrate proficiency with a number of professional and analytical competencies. The purpose of this study was to determine the impact of Hands and Health at Home, an intergenerational hand-training program, as a gerontology-focused educational experience. In this program, upper level and graduate pre-health students were trained to deliver hand-based exercises to homebound older adults twice a week for 8 weeks. Students completed the Geriatric Attitudes Scale (GAS), Coping Self-Efficacy Scale (CSES), and a program evaluation survey. Forty students completed Hands and Hand at Home across three semesters. After the program, scores on the GAS (p< 0.05) and CSES (p< 0.05) significantly improved. Feedback about the program was overwhelmingly positive, where students indicated high satisfaction, said they would recommend the program to a friend, and felt prepared for their home training sessions. When asked to express any skills learned during the program, students often reported communication, teamwork, compassion, confidence, and creativity. The results from Hands and Health at Home demonstrate that this intergenerational service-learning program was a mutually beneficial way to deliver hand training to homebound older adults while also providing valuable hands-on experience for pre-health students. The program also resulted in reductions in ageist attitudes, improved self-efficacy, and the development of several important pre-health competencies, underscoring the need to offer more intergenerational and gerontology-based educational opportunities.

